# Prevalence of suicidal ideation and suicide attempts among refugees: a meta-analysis

**DOI:** 10.1186/s12889-022-13029-8

**Published:** 2022-04-01

**Authors:** Elisa Haase, Antje Schönfelder, Yuriy Nesterko, Heide Glaesmer

**Affiliations:** 1 Department of Clinical Psychology and Psychotherapy, University of Leipzig, Neumarkt 9-19, 03081 Leipzig, Germany; 2 Department of Medical Psychology and Medical Sociology, University of Leipzig, Philipp-Rosenthal-Str. 55, 04103 Leipzig, Germany

**Keywords:** Suicidal ideation, Suicide attempts, Refugees, Mental health, Asylum-seekers, Prevalence

## Abstract

**Background:**

Suicidal ideation and attempts are one of the most serious mental health problems affecting refugees. Risk factors such as mental disorders, low socio-economic status, and stressful life events all contribute to making refugees a high-risk group. For this reason, this meta-analysis aims to investigate the prevalence of suicidal ideation and attempts among refugees in non-clinical populations.

**Method:**

We searched *PubMed, Web of Science, PubPsych*, and *PsycInfo* for articles reporting (period) prevalence rates of suicidal ideation and attempts. Inclusion criteria were the population of refugees or asylum seekers (aged 16 years and older), assessment of the prevalence of suicidal ideation and attempts in empirical studies in cross-sectional or longitudinal settings, written in English, and published by August 2020. Exclusion criteria were defined as a population of immigrants who have lived in the host country for a long time, studies that examined children and adolescents younger than 16 years, and research in clinical samples. Overall prevalence rates were calculated using Rstudio.

**Results:**

Of 294 matches, 11 publications met the inclusion criteria. The overall period prevalence of suicidal ideation was 20.5% (CI: 0.11–0.32, I^2^ = 98%, *n* = 8), 22.3% (CI: 0.10–0.38, I^2^ = 97%, *n* = 5) for women, and 27.7% for men (CI: 0.14–0.45, I^2^ = 93%, *n* = 3). Suicide attempts had an overall prevalence of 0.57% (CI: 0.00–0.02, I^2^ = 81%, *n* = 4).

**Conclusion:**

There is a great lack of epidemiological studies on suicidal ideation and attempts among refugees. The high prevalence of suicidal ideation indicates the existence of heavy psychological burden among this population. The prevalence of suicide attempts is similar to that in non-refugee populations. Because of the large heterogeneity between studies, the pooled prevalence estimates must be interpreted with caution. The results underline the need for systematic and standardized assessment and treatment of suicidal ideation and attempts.

**Supplementary Information:**

The online version contains supplementary material available at 10.1186/s12889-022-13029-8.

## Background

Approximately 800,000 people commit suicide each year, making suicide one of the leading causes of death worldwide [1]. Thus, suicide is a global and complex public health problem. There are distinct differences in the development of suicidal ideation and attempts associated with gender, age, geographic region, and socio-political conditions [2]. People who live in war zones, experience armed conflicts, persecution, displacement, traumatization, social isolation, and somatic and mental illnesses are particularly at risk [1]. Forced displacement and seeking asylum often involve dangerous travel, separation from family and friends, inhumane living conditions, and fear of being detainment and death. In addition, post-migration stressors such as perceived discrimination, restricted access to health care, education and/or work, etc. are further problems refugees face after they arrived in secure host countries [3]. Accordingly, refugees and/or asylum-seekers are at high risk for any kind of suicidal ideation and attempts before, during, and/or after flight [4].

Due to the multitude of stressful events that most refugees experience resulting from having to leave their homes, the likelihood of them developing mental disorders is substantially elevated. The most common disorders are depression, anxiety disorders, post-traumatic stress disorder, and somatization disorders [5–8]. Individuals with mental disorders and stressful life events are more likely to suffer from suicidal ideation and to attempt suicide.

## Suicidal ideation

The global lifetime prevalence of suicidal ideation is 9.2% across 17 countries in Africa, Asia and the Pacific, North America, Europe, and the Middle East; *n* = 84,850) [9], 3.1–12.0% in developing countries, and 3.0%-15.9% in developed countries. Twelve-month prevalence estimations across 21 countries are 2.0% for developed countries (e.g. France, US, Germany, Israel, Japan) and 2.1% for developing countries (e.g. Brazil, Bulgaria, India, Lebanon, Nigeria, South Africa) (*n* = 108,705) [10]. In a review of 86 studies worldwide, Liu et al. [11] showed that the twelve-month prevalence of passive suicidal ideation (e.g. “thoughts to be better off dead”, “desire to be dead”) was 5.8% and lifetime prevalence was 10.6% in the general population.

General risk factors for suicidal ideation include: previous suicidal ideation, feelings of hopelessness, depression, anxiety, abusive experiences of any kind [12], somatic pain [13] and female gender [9, 14].

Prevalence data on suicidal ideation and attempts in refugee populations are scarce. Studies on suicidal ideation show significantly higher rates among refugees compared to non-refugee populations. In a study on suicidal ideation (experienced in the month before responding to the survey) among refugees from Libya, Sudan, Congo, etc., in Nigeria, 27.3% (*n* = 444) of refugee participants reported suicidal ideation, a significant difference compared to 17.3% (*n* = 527) of that host country’s residents (*n* = 527) [15]. Other studies conducted in initial reception centres for asylum-seekers reported prevalence for suicidal ideation in the previous two weeks of 33.9% (*n* = 510) in refugees from Afghanistan and Syria [16] in Sweden, and 5.6% (*n* = 209) in Germany [17].

Based on the studies described above, it is safe to say there is a great deal of variation in the prevalence of suicidal ideation. Factors related to suicidal ideation in refugees are: inability to provide for family (e.g. work disability; 21), low levels of social support, anxiety, depression [18, 19], high mental distress [16], low quality of life [15] and insecure visa status [20].

## Suicide attempts

A study by Nock et al. [9] analyzed cross-national sample data (17 developed and developing countries) on suicide attempts; they found a lifetime prevalence of 2.7%. The statistics in developing countries (0.7%—4.7%) and developed countries (0.5%—5.0%) are similar. The likelihood of those who experience suicidal ideation ever attempting suicide is estimated to be 29.0% [9].

Data from the *World Mental Health Survey* (WMH, conducted from 2001–2007, *n* = 108,705) identified sociodemographic characteristics (age, sex, education, family income, marital status, and employment), parental psychopathology (major depressive episode, panic disorder, generalized anxiety disorder, substance use disorder, antisocial personality disorder), childhood adversities (childhood losses and family dysfunction, major physical illness), respondent's past suicidality and respondent's mental health status in the past 12 months as predictors of suicide attempts [10]. Sundvall et al. [21] compared asylum seekers and Swedish citizens (all people with a Swedish personal identity number) in terms of factors that influenced recent suicide attempts. Swedish citizens had different risk factors and disease patterns compared to asylum seekers, among whom suicide attempts appear to be driven by the asylum decision process, previous mental health problems, and pre-migration stressors. Suicide attempts have the highest power in predicting future suicides [22] and therefore require special attention as a key risk factor.

There are several studies investigating suicide attempts in refugees. Incidence rates in refugee camps in Thailand range from 30–35 per 100,000 refugees in 2014–2016 [23]. In Sweden, three cohorts (1999–2009) of the entire Swedish population were studied (approximately 5 million in each cohort, 3.3–5.0% refugees): hazard ratios regarding suicide attempt in refugees, compared to Swedish-born, range from 0.38–1.25 depending on country of birth. The results were either not significant or refugees showed a lower risk of suicide attempts [24, 25] report 0.8% (*n* = 129) suicide attempts during the past year compared to 0.2% (*n* = 1,290) in Korean nationals.

In his systematic review and meta-analysis, Amiri [26] summarized the prevalence of immigrants' suicidal ideation (16%, CI: 0.12–0.20, I^2^ = 99.4%) and suicide attempts (6%, CI: 0.05–0.08, I^2^ = 98.0%). His analyses include prevalence data on refugees as well as other groups of immigrants. In our meta-analysis, we focus on refugee samples only. Because there is considerable evidence of significantly elevated prevalence rates of mental disorders among refugees compared with native-born and non-refugee immigrant populations (e.g. 28), it does not seem reasonable to compute overall prevalence among both refugees and other immigrant groups together. Therefore, this meta-analysis only includes studies in non-clinical populations and studies examining refugees (and asylum seekers) shortly after their arrival in host countries or refugee camps.

## Methods

### Search strategy and selection criteria

Inclusion criteria as well as the methods of analysis were specified and documented at the beginning of the literature search. For this, we used the *PICOS* scheme (*[P]* patient population or disease to be treated, *[I]* interventions or exposures, *[C]* comparison group, *[O]* outcome or endpoint, and *[S]* study design chosen) to structure the literature search [27]. The criteria are shown in Table 1.

**Table 1:** Inclusion and exclusion criteria according to PICOS scheme (O'Connor et al., 2008)

Inclusion criteria	
***P****opulation*	refugees^a^ or asylum seekers^b^, age 16 years and older
***I****ntervention*	not required
***C****omparison*	not required
***O****utcome*	detection of suicidal ideation and attempts in refugees and asylum seekers
***S****tudy type*	empirical studies on the prevalence of suicidal ideation and suicide attempt
***S****tudy design*	cross-sectional or longitudinal studies on the prevalence of suicidal ideation and suicide attempts; written in English language; published until August 2020
**Exclusion criteria**	
• Immigrants, living in the host country for a long time (first, second generation immigrants) • Studies focused on refugees or asylum seekers under the age of 16 years • Studies conducted in clinical populations	

^a^ A refugee is an individual who is “owing to a well-founded fear of being persecuted for reasons of race, religion, nationality, membership of a particular social group or political opinion, is outside the country of his nationality and is unable to, or owing to such fear, is unwilling to avail himself of the protection of that country” (https://www.unhcr.org/protect/PROTECTION/3b66c2aa10.pdf)

^b^ An asylum-seeker is an individual who is seeking international protection. In countries with individualized procedures, an asylum-seeker is someone whose claim has not yet been finally decided on by the country in which he or she has submitted it. Not every asylum-seeker will ultimately be recognized as a refugee, but every refugee is initially an asylum-seeker.” (UNHCR, 2016)

We followed the *PRISMA (Preferred Reporting Items for Systematic reviews and Meta-Analyses)* guidelines for reporting systematic reviews and meta-analysis [28].

All studies published up through August 2020 and written in English were considered for the analysis. We searched *PubMed, Web of Science, PubPsych,* and *PsycInfo* for articles reporting on prevalence rates of suicidal ideation and suicide attempts. The following search string was used: [“Suicidal ideation” and “asylum seekers” or “refugees”], [“suicide attempt” and “asylum seekers” or “refugees”], and [“suicidal behavior” and “asylum seekers” or “refugees”], with and without the term [and “prevalence”].

First, the articles were selected by title and abstract (E.H., P.A.). The screening of the abstracts was performed independently and simultaneously by a student assistant (P.A.) and a research assistant (E.H.). Next, the full texts were screened (E.H.). The screening of the full articles was conducted exclusively by the research assistant (E.H.). In case of ambiguities, the senior author (H.G.) was consulted and a joint decision was made (E.H., H.G.). Articles were chosen for inclusion in the meta-analysis based on the inclusion and exclusion criteria defined beforehand (tbl.1) and the completeness of the data. If the same sample was reported on in several articles, we selected the article with the most comprehensive information. In some cases, we contacted the authors of the articles to get more information on their data [16, 17, 20, 29]. Only two of them responded [17, 29]. Führer et al. (2016) provided a detailed description of the frequencies of responses to questionnaire items related to suicidal ideation (differentiated by gender), as mentioned in Table 2. Meyerhoff et al. (2020) shared with us the frequencies of suicidal ideation and suicide attempts broken down by gender.

**Table 2:** Quality Assessment of included studies (EPHPP)

Author and year of publication	Selection bias	*Rating*	Study design	*Rating*	Data collection method	*Rating*	*Rating total*
Akinyemi et al. (2015) [15]	random selection	*moderate*	cross-sectional, cluster sampling	*strong*	Mini-International Neuropsychiatric Interview (MINI) *(In the past month did you think that you would be better off dead or wish you were dead?; In the past have you thought about killing yourself?),* last month	*strong*	***moderate***
Alley (1982)	random selection	*moderate*	statewide survey, cross-sectional	*moderate*	-	*weak*	***moderate***
Bhui et al. (2003) [30]	random selection	*strong*	cross-sectional	*strong*	Beck Depression Inventory (BDI) *(I don't have any thoughts of killing myself.(0),* *I have thoughts of killing myself, but I would not carry them out. (1), I would like to kill myself. (2),* *I would kill myself if I had the chance. (3))*, last two weeks	*strong*	***strong***
Cochran et al. (2013) [31]	random selection	*moderate*	cross-sectional	*strong*	Interview, *“[…] asked if they had ever expressed suicidal* *ideation (i.e., ever thought seriously about committing suicide in their lifetimes)”*, lifetime and last month	*moderate*	***moderate***
Falb et al. (2013) [32]	random selection	*strong*	survey, cross-sectional	*strong*	non-standardized interview following guidelines of the Reproductive Health Toolkit for Conflict-Affected Women, last month	*moderate*	***strong***
Führer et al. (2016) [17]	random selection	*moderate*	survey, cross-sectional	*strong*	Hopkins-Symptom-Checklist-25 (HSCL-25) *(Thoughts of ending your life: Not at all (0), A little (1), Quite a bit (2), Extremely (3))*, last week	*strong*	***strong***
Leiler et al. (2019) [16]	random selection	*moderate*	survey, cross-sectional	*strong*	PHQ-9 *(Thoughts that you would be better off dead, or of* *hurting yourself: Not at all Several (0), several days (1), More than half the days (2), Nearly every day (3)),* last two weeks	*strong*	***strong***
Meyerhoff et al. (2020) [29]	random selection	*moderate*	cross-sectional	*strong*	Beck Scale for Suicidal ideation (BSS) *(assesses various aspects of suicidal ideation an attempt on a 3-point scale),* last week	*strong*	***moderate***
Nickerson et al. (2019) [20]	random selection	*moderate*	cross-sectional, snowball-sampling	*strong*	PHQ-9 *(Thoughts that you would be better off dead, or of* *hurting yourself: Not at all Several (0), several days (1), More than half the days (2), Nearly every day (3)),* last two weeks	*strong*	***strong***
Rahman et al. (2003) [33]	random selection in primary health care	*moderate*	survey, cross-sectional	*moderate*	Self-Reporting Questionnaire (SRQ-20) *(Has the thought of ending your life been on your mind?: yes/no)*, last month	*moderate*	***moderate***
Sohn et al. (2019) [25]	random selection	*moderate*	survey, cross-sectional	*moderate*	the Korea National Health and Nutrition Examination Survey (KNHANES) *Interview*, last year	*moderate*	***moderate***

## Data extraction and analysis

We formulated a data extraction form to systematically extract sample size and gender distribution, prevalence of suicidal ideation, and prevalence of suicide attempts separately (if possible, for women, men, and gender-diverse), which can be found in the 2nd to 4th columns of Table 2.

The analyses were performed with *RStudio* [34], using the packages “meta” [35] and “metafor” [36, 37]. Transformation methods were used to avoid inappropriate weighting of studies with small or large prevalence. In the present case, data was transformed with an arcsine algorithm to stabilize sampling variances [38]. The data were then back-transformed to see the output for the true summary proportion and its 95% confidence interval. A random effects model was selected for pooling the data using the restricted DerSimonian-Lairs estimator. We chose a random effects model because we expected variation across studies (heterogeneity), for example, in the methods used and their samples. To test for heterogeneity, we used I^2^ to quantify it, and Cochrane ‘s Q to assess statistical significance. We also used forest plots to check for asymmetry. Outlier analyses were also carried out. The adjustment for outliers is only useful if it reduces heterogeneity and/or if there is a theoretical reason (e.g. population, method) why studies can be identified as outliers [37, 39]. Neither of these aspects were met in the present meta-analysis, hence we did not adjust for outliers.

Funnel plots were performed to explore the publication bias. Moreover *rank correlation test* [40] and *Egger's regression test* [41] were used to assess publication bias.

## Results

**Fig. 1:**
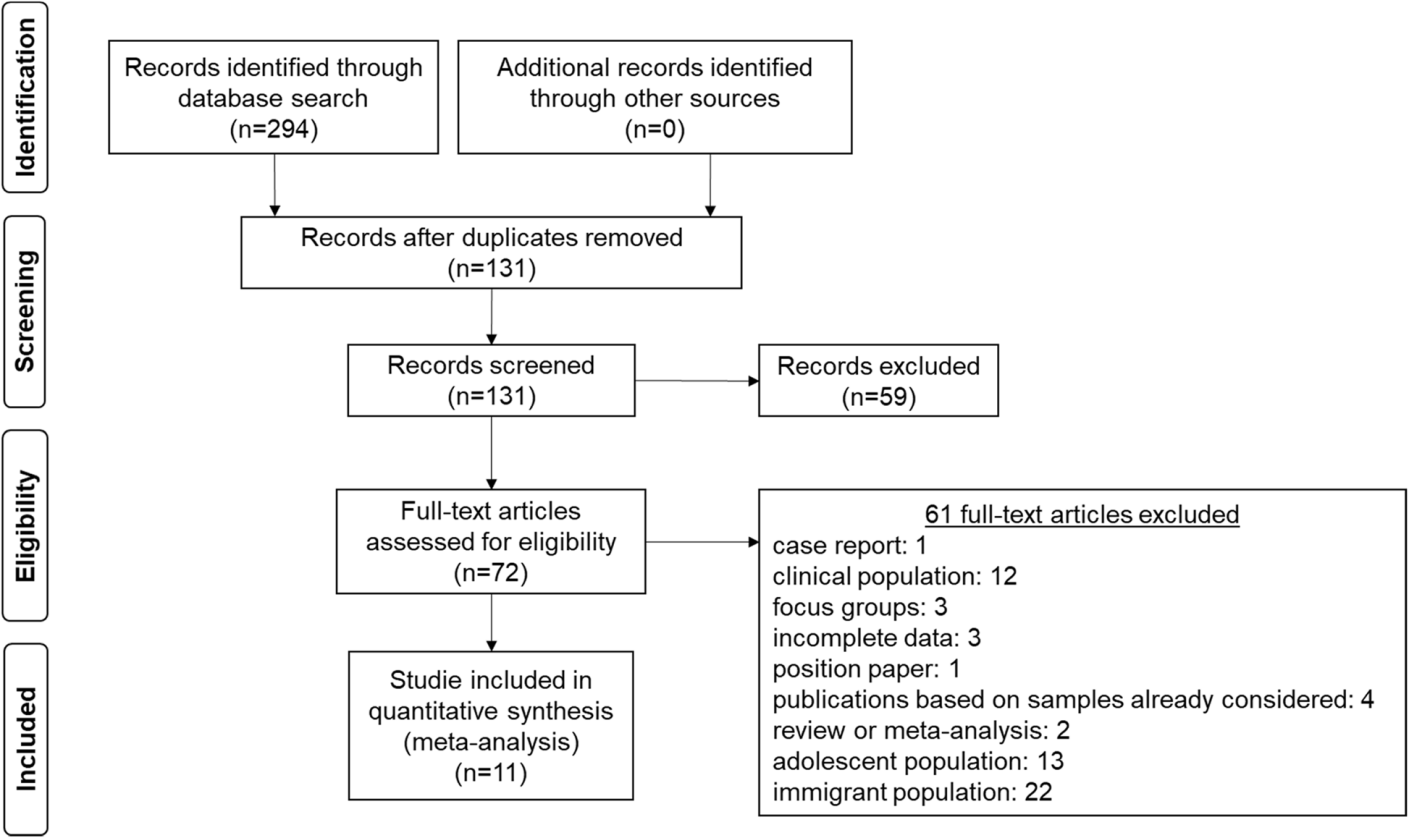
Flow diagram to reconstruct study selection

The search of the digital platforms resulted in 294 hits that were screened and systematically examined for the defined criteria. An overview of this process can be found in Fig. 1.

After excluding all studies that did not match inclusion criteria, did match the exclusion criteria, or were duplicates, 11 studies could be included in the meta-analysis. Detailed information on these studies are presented in Table 2. Not all 11 studies could be used in each analysis because not every study reported both suicidal ideation and suicide attempts and/or reported prevalence separated by gender (suicidal ideation: *n* = 8; suicidal ideation in women: *n* = 5, suicidal ideation in men: *n* = 3; suicide attempts: *n* = 4). The sample size, quality of data collection, and assessment methods (interviews, self-report questionnaires, and individual items) widely varied among the included studies. The periods of time for which suicidal ideation was examined ranged from one week (*n* = 2), two weeks (*n* = 4) and four weeks (*n* = 2). For men, the distribution was as follows: one week (*n* = 1), two weeks (*n* = 1), and four weeks (*n* = 1); and for women, one week (*n* = 1), two weeks (*n* = 1), and four weeks (*n* = 3). The periods of time for suicide attempts ranged from one week (*n* = 1), two weeks (*n* = 1), and four weeks (*n* = 1) to one year (*n* = 1) (in one article there was no indication of the survey period).

Table 2 gives an overview of the prevalence of suicidal ideation across the studies. For suicidal ideation, the studies ranged from 2.13%—34.4%, and for suicide attempts from 0.14–7.55%.

## Assessment of methodological quality

To assess the quality of the selected studies we used the factors of the quality assessment tool *EPHPP* (*Effective Public Health Practice Project*; [42]) that are applicable to this analysis (selection bias: representative target sample, participation rate in percent; study design: type of study design, method of randomization; data collection method: validity and reliability of tools). For this purpose, the individual sections were classified as strong (1), moderate (2), and weak (3) in quality according to a predefined evaluation key [43]. The overview of this quality assessment can be found in Table 3.

**Table 3:** Study characteristics

Author and year of publication	Suicidal ideation	Suicide attempt	Sample size	Gender distribution	Age distribution	Population	Country
**Akinyemi et al. (2015) **[15]	121 (27.3%) female: 66 male: 55	-	444	female: 263 male: 181	18 and older mean: 34.7 (SD = 12.8)	refugees (Liberians, Sierra Leonans, Sudanese, Congolese, Eritreans)	Oru-Ijebu, South-West Nigeria
**Alley (1982)**	-	6 (0.14%) female: 4 male: 2	4.192	-	17–52	Indochinese refugees	Utha, USA
**Bhui et al. (2003) **[30]	62 (34.4%) female: 24 male: 38	-	180	female: 89 male: 91	mean: 40.4 range: 20–88	Somali refugees	Greenwich/London, GB
**Cochran et al. (2013) **[31]	13 (lifetime) (3.07%) 9 (last month) (2.13%)	1 (0.24%)	423	female: 202 male: 221	18 and older	Buhatan refugees	Arizona Georgia, New York and Texas, USA
**Falb et al. (2013) **[32]	female: 63 (7.43%)	-	female: 848	only women	15–49 mean: 32.12 (SD = 8.42)	female refugees in a partnership	Thai–Burma border, Myanmar & Thailand
**Führer et al. (2016) **[17]	33 (15.79%)^a^ female: 6 male: 25 others: 1 missing:1	-	209	female: 24 male: 177 others: 3 missing:5	16 and older	asylum-seekers	Halle, Germany
**Leiler et al. (2019 **[16]		173 (33.92%)	510	female: 136 male: 367 others: 7	18 and older	refugees (Afghanistan, Syria)	Jämtland-Härjedalen county, Sweden
**Meyerhoff et al. (2020) **[29]	4 (6.67%)	4 (7.55%)	60 (ideation)/ 53 (attempt)	ideation: female: 29 male: 31 attempt: female: 22 male: 31	18–65 mean women: 43.7 (SD = 10.5) mean men: 38.2 (SD = 10.8)	Buhatan refugees	greater Burlington, Vermont region, USA
**Nickerson et al. (2019 **[20]	102 (39.38%)	-	259	female: 81 male: 178	18 and older, mean: 38.11 (SD = 11.8)	refugees or asylum-seekers (Iraq, Syria, Iran, Sri Lanka, Afghanistan) with no secure visa	Australia
	154 (18.64%)	-	826	female: 384 male: 442		refugees or asylum-seekers (Iraq, Syria, Iran, Sri Lanka, Afghanistan) with secure visa	
**Rahman et al. (2003) **[33]	female: 96 (32.32%)	-	female: 297	only women	mean: 28.2 (SD = 7.3)	female refugees which are mothers	Shamshatu and Shalman; Afghanistan
**Sohn et al. (2019) **[25]	-	1 (0.78%)	129	female: 93 male: 36	< 31: 44 31–40: 50 41–50: 27 > 51: 8	refugees and asylum seekers (Nigeria, Ethiopia, Liberia, Yemen, Egypt)	Seoul and Gyeonggi province, Korea

^a^ After correspondence with the authors, we received the detailed frequency tables on suicidal ideation and classified those who responded with 1 (‘a little”), 2 (‘quite a bit’) to 3 (‘extremly’) as individuals with suicidal ideation. The classification thus differs from that in the paper

**Fig. 2:**
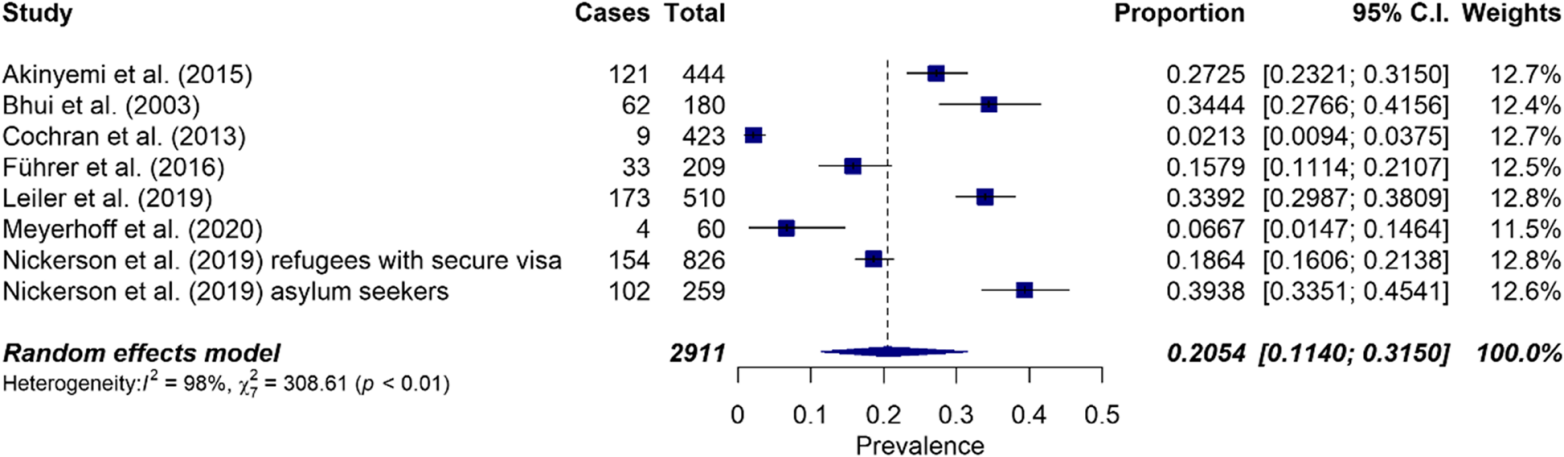
Forest plot on the prevalence of suicidal ideation (*n* = 8)

The overall prevalence of suicidal ideation was 20.5% (CI: 0.11–0.32, *n* = 8). Statistical assessment of heterogeneity revealed I^2^ = 98% and Cochrane's Q = 308.61, *p* < 0.0001. Forest plots were used to graphically test heterogeneity (Fig. 2). There was significant heterogeneity between studies. For this reason, subgroup analyses would have been useful, but could not be performed due to the small number of studies.

**Fig. 3:**
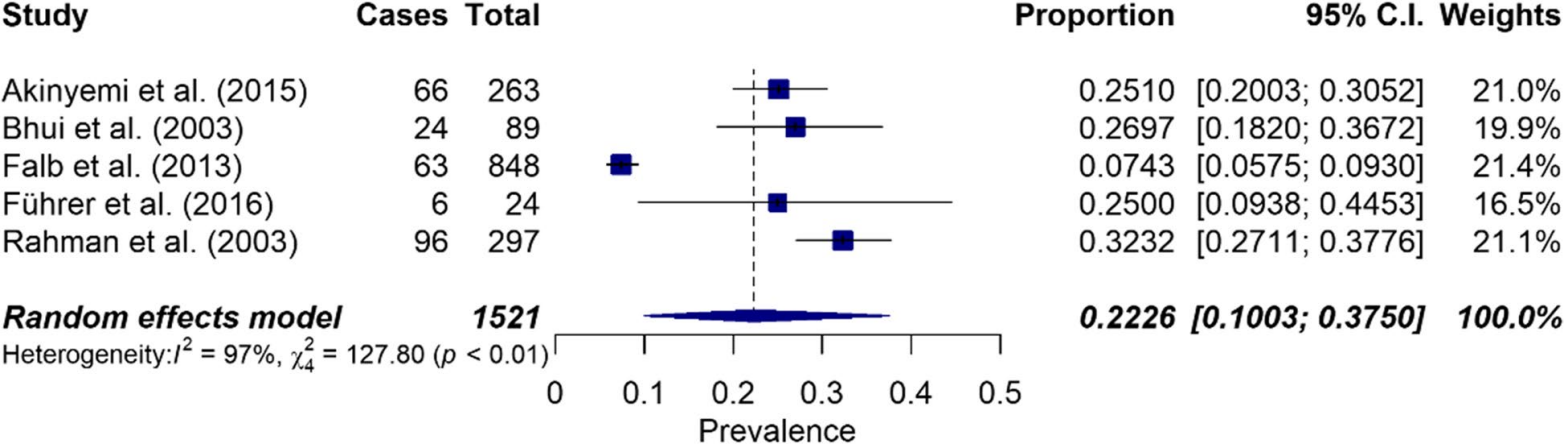
Forest plot on the prevalence of suicidal ideation in women (*n* = 5)

**Fig. 4:**
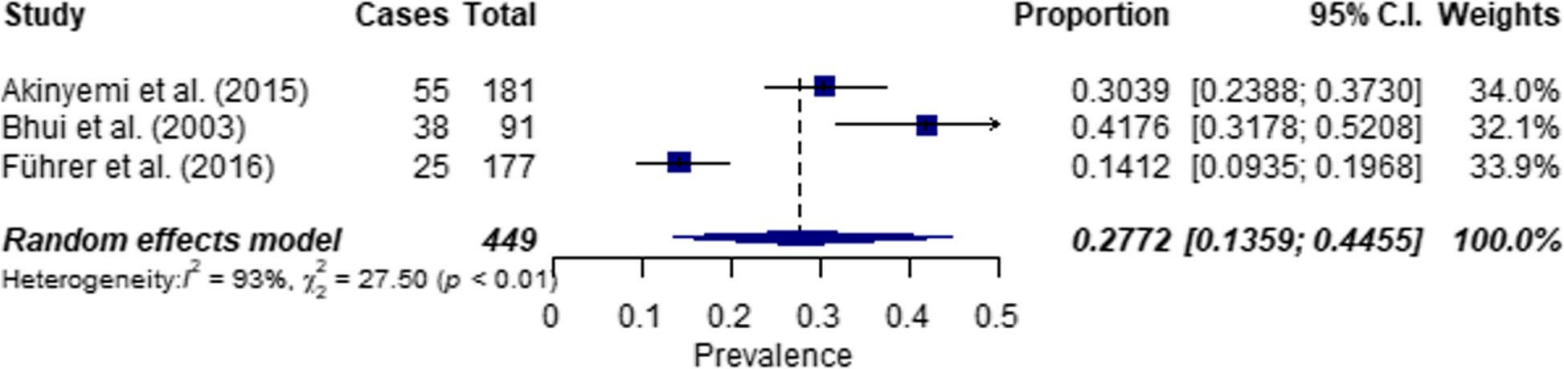
Forest plot on the prevalence of suicidal ideation in men (*n* = 3)

For suicidal ideation in women, an overall prevalence of 22.3% (CI: 0.10–0.38, *n* = 5) was calculated, but high heterogeneity exists in this subpopulation (I^2^ = 97%, Q = 127.80, *p* < 0.0001) (see Fig. 3). The overall prevalence of suicidal ideation among men was 27.7% (CI: 0.14–0.45, *n* = 3) with high significant heterogeneity (I^2^ = 93%, Q = 27.4972, *p* < 0.0001) (see Fig. 4).

**Fig. 5:**
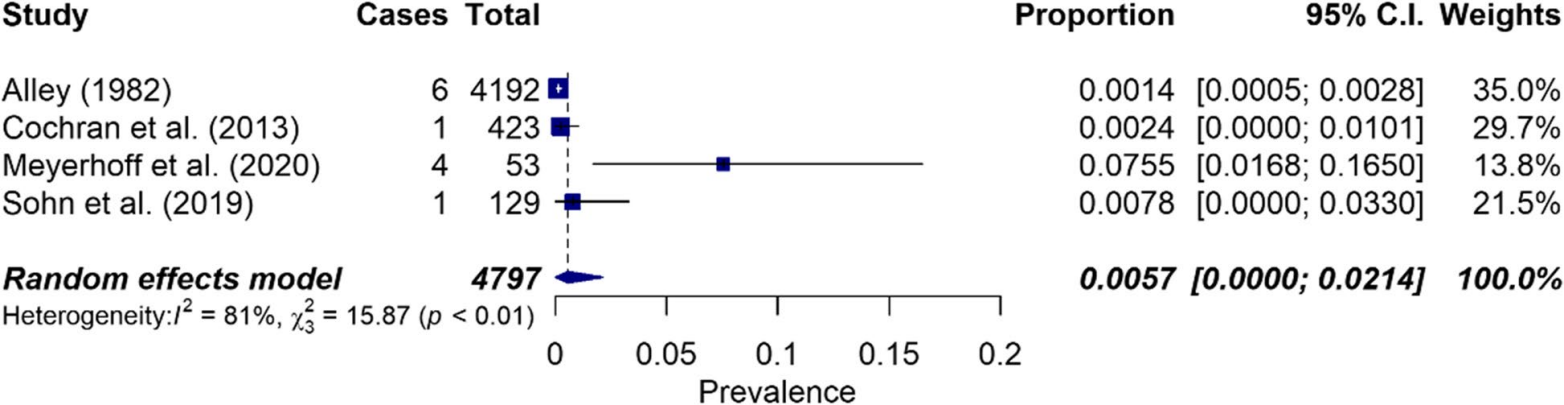
Forest plot on the prevalence of suicide attempts (*n* = 4)

Suicide attempts had an overall prevalence of 0.57% (CI: 0.00–0.02, *n* = 4). Heterogeneity was significantly high (I^2^ = 81%, Q = 15.88, *p* < 0.0012) (see Fig. 5).

Publication bias can likely be ruled out, as the Begg and Mazumdar [40] rank correlation test result, *p* = 0.0833, was not significant. In parallel, Eggers regression test [41], *p* = 0.0020, was significant. In addition, a funnel plot was created to test for publication bias (see Appendix; figure A1), indicating funnel plot asymmetry and high heterogeneity.

## Discussions

The two meta-analyses presented here calculated overall prevalence of suicidal ideation and attempts in refugees and asylum seekers. Based on the eight studies included a prevalence of 20.5% (range: 2.13%—34.4%) was found for suicidal ideation across both genders. When distinguished by gender, a prevalence of 22.3% (*n* = 5) was found for women and 27.7% (*n* = 3) for men. The slightly higher prevalence compared to the total population is due to the fact that not all of the 11 studies offered data separated by gender. Two factors were at work here: 1) fewer studies could be used to calculate the gender-specific prevalence; and 2) two of the studies included only women [32, 33]. In determining an overall prevalence of suicide attempts, it was possible to include four studies, yielding a prevalence of 0.57% (range: 0.14%—7.55%).

Compared to global prevalence data [9–11], there is a higher prevalence of suicidal ideation among refugees. Direct comparisons are difficult, however, because global prevalence data are often recorded as lifetime or 12-month prevalence rates. That said, in the studies considered here, periodic prevalence rates were reported including the 1–4 weeks prior to when the surveys were administered (see Table 3). Only Cochran et al. [31] reported a lifetime prevalence of suicidal ideation among Bhutanese refugees in the United States of 3% (*n* = 423). This corresponds to the lower range of previously reported global prevalence rates [9].

In looking at prevalence rates for suicide attempts, we found numbers similar to those reported in cross-national studies [9]. Again, comparison is difficult because the data on suicide attempts in the included studies reflects a variety of time periods ranging from one week to one year (see Table 3).

With one exception [17], studies on refugee populations report substantially higher prevalence rates of suicidal ideation compared to studies examining non-refugee populations or the general population. All in all, the results emphasize previous findings of increased psychological distress among refugees being associated with higher rates of suicidal ideation and suicide attempts [4]. Amiri [26] included studies done with various immigrant groups and refugees in his review without distinguishing between them (n = 29). But as mentioned above, people who leave their country of origin do so for a variety of reasons; for refugees in particular, the flight is more often accompanied by numerous traumatic events. In addition, it must be taken into account that immigrants who have already lived and worked in their new host country for several years, are exposed to different stressors than people who have been on the run for the past few months or years, and have just arrived in a host country and/or have an uncertain asylum status (pre-, peri- and post-migration factors). On the other hand, there may be an increased vulnerability as a result of pre- and peri-migration stressors, which can result in mental disorders and suicidal ideation.

Studies in immigrant populations in different European countries (e.g. Germany, Sweden) show 1^st^ generation immigrants having less suicidal ideation than host country residents or 2^nd^ generation immigrants (e.g. [44, 45]). This phenomenon can be explained by the *Protective Culture Model* (e.g. [45]), which assumes that some protective factors against suicide might be linked with culture of origin (e.g. religious beliefs, stable family bonds, social support). This reduces the stress of acculturation. However, this protection decreases with time spent in the host country, resulting in increased suicidal ideation and attempts in the second generation. Factors influencing suicidality among immigrants are acculturation stress, experiences of discrimination, cultural differences, and environmental factors, among others (for an overview, see [46]). Also worth considering are post-migration stressors such as socioeconomic factors, social and interpersonal circumstances, and stressors related to the asylum process and immigration policies [15, 47], which can be burdensome. Considering these differences between refugees and/or asylum seekers compared to immigrants (immigrants or former refugees living in their host country for some time), it is not reasonable to equate their prevalence rates of suicidal ideation and attempts. The danger of doing so is that it could result in underestimating the general risk of suicidal ideation and suicide attempts among refugees and asylum seekers.

## Strengths and limitations

The strengths of this analysis lie in the methodology that was used, which is based on current research standards [27, 35, 38, 48]. The screening of abstracts was done independently and simultaneously by a student assistant (P.A.) and a research assistant (E.H.), but this was not possible during the screening of articles’ full texts due to organizational reasons, which must be considered as a methodological deficiency.

All studies were rated "moderate" or "strong" in quality assessment (tab. 3). In some cases, however, the sample sizes were relatively small (e.g. 20, 28, 32, 49), which could have an impact on external validity. Another limitation is the small number (*n* = 11) of studies that could be integrated into the meta-analysis after reviewing articles’ full texts. As we could not include all 11 studies in one analysis due to the data situation described in the results, a meta-analysis was calculated on suicidal ideation (*n* = 8; women: *n* = 5, men: *n* = 3) and on suicide attempts (*n* = 4) separately. This reduced the number of studies that could be considered in each analysis. For this reason, the generalizability of the results is of a rather limited nature. Generalization of findings on suicide ideation among women may be further compromised by the fact that very specific groups of women were studied, such as female refugees in a partnership [32] or exclusively mothers [33]. These subgroups may be subject to additional protective or risk factors that were not considered in the research.

We included studies that were not initially designed as epidemiological studies, but in which descriptive data of the periodic prevalence of suicidal ideation and attempts among refugees and asylum seekers were recorded. Furthermore, no gray literature search was conducted, so there may be unpublished research that was not included.

In view of the different survey methods (mainly self-report) used to record suicidal ideation and suicide attempts, of the included studies, the appropriateness of calculating pooled prevalence estimates could be questioned as well as internal validity. In general, to compare test results in different cultural groups, equivalence in language and construct validity must be taken into account.

Regarding the Publication Bias, Eggers regression test [41], *p* = 0.0020, was significant, but the explanatory power is only moderate in samples comprised of fewer than 25 studies. The funnel plot (see Appendix; Figure A1) also indicated asymmetry and high heterogeneity. Because prevalence data do not describe positive or negative effects (e.g. effectiveness of an intervention), low prevalence rates are no less likely to be published than high ones. Therefore, classical methods for assessing publication bias in meta-analyses of observational studies might be of limited use [37].

Nevertheless, because of high heterogeneity between studies, the pooled prevalence estimates should be interpreted with caution. In this meta-analysis, the high heterogeneity could be related to the fact that 10 different assessment methods were used, there was a significant variance in host countries and countries of origin, and therefore cultural background and attitudes toward suicidality. Subgroup analyses would have been necessary to actually define the variance between studies as a function of the factors just mentioned. These, however, could not be performed in the present meta-analysis due to the small number of studies included (there must be at least 10 studies to perform a subgroup analysis, [49]). Thus, future research in the same field of interest should focus in more detail on subgroups regarding differences in host countries, cultural characteristics such as religion or attitudes toward mental disorders, country of origin, and flight duration and/or route, etc.

## Conclusions

The results of the meta-analysis provide evidence of a high prevalence of suicidal ideation in the considered population. The prevalence of suicide attempts is similar to the non-refugee population. Only a few studies could be included, which showed a large heterogeneity, so that a generalization of the results is not possible. In this context, it is worth noting that only few studies to date have systematically and adequately asked about suicidal ideation and attempts in the population of refugees and asylum seekers. Therefore large non-biased epidemiological studies are needed to determine robust prevalence estimates. In addition, longitudinal studies would be helpful for tracking the persistence of suicidal ideation and repeated suicide attempts in refugees and asylum seekers.

Based on the results, it could be useful to screen newly arriving refugees for suicidal ideation and attempts as well as mental disorders in the initial accommodation facilities when they seek counseling or medical care. In this way, timely and appropriate help could be provided.

When screening for suicidal ideation and attempts, both culturally sensitive and standardized diagnostic procedures should be used that take psychometric properties (ensuring psychometrically validated language versions and cultural equivalence of language versions) into account. Respecting these factors is key to ensuring a sensitive approach to the respondents and the comparability of future studies.

To address the issue of suicidality prevention among refugees and asylum seekers, risk factors for suicidal ideation, attempts, and completed suicides need to be explored. To do so, a better understanding of clinical, psychological, cultural, and sociological factors is important and would consequently help in identifying high-risk individuals and providing first aid or treatment (e.g., counseling, psychotherapy, acute psychiatry). Building on initial research in refugee populations addressing specific risk factors for suicidal ideation (e.g. [16, 18–21, 24]), it would be of great interest to further explore factors in order to identify general risk factors. In this context, the information could be used to develop and expand interventions to address suicidality in refugees.

## Supplementary Information

**Additional file 1.**

## Data Availability

All data generated or analyzed during this study are included in this published article [and its supplementary information files].

## Abbreviations

PICOS scheme: ([P] patient population or disease to be treated, [I] interventions or exposures, [C] comparison group, [O] outcome or endpoint, and [S] study design chosen)
PRISMA: (Preferred Reporting Items for Systematic reviews and Meta-Analyses)
EPHPP: (Effective Public Health Practice Project)
