# Supplementary figures and images for: Prevalence of suicidal ideation and suicide attempts among refugees: a meta-analysis
Source: BMC Public Health. 2022 Apr 1;22:635. doi: 10.1186/s12889-022-13029-8 (PMC8976302; doi:10.1186/s12889-022-13029-8)

**Appendix**

**
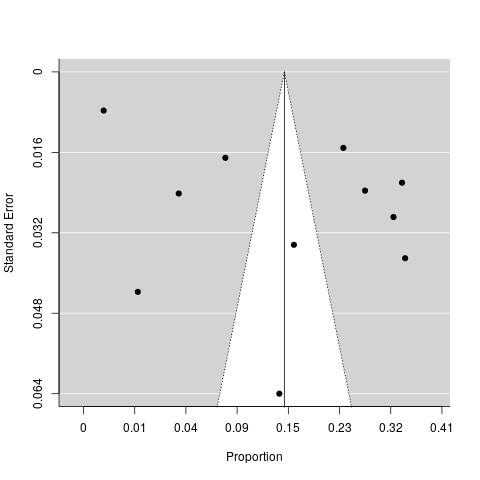
**

**Figure A1) Funnel plot - publication bias**

Supplement: Supplementary file 1 — Additional file 1. [file 12889_2022_13029_MOESM1_ESM.docx]
